# 196. Does Cefepime Provide an Advantage over Ceftriaxone in the Treatment of Bloodstream Infections due to *Escherichia coli, Klebsiella pneumoniae* group, *Klebsiella oxytoca,* and *Proteus* spp. if CTX-M is not Detected by Rapid Molecular Testing?

**DOI:** 10.1093/ofid/ofad500.269

**Published:** 2023-11-27

**Authors:** Katie Lynn Hammer, Alyssa Hamm, Stephanie N Welch, Jordan R Smith, Leigh Ann Medaris, Michael S Boger, Michael S Boger, Julie E Williamson

**Affiliations:** Atrium Health, Mount Pleasant, South Carolina; Atrium Health Carolinas Medical Center, Charlotte, North Carolina; Atrium Health, Mount Pleasant, South Carolina; High Point University, High Point, North Carolina; Atrium Health, Mount Pleasant, South Carolina; Atrium Health, Mount Pleasant, South Carolina; Atrium Health, Mount Pleasant, South Carolina; Atrium Health, Mount Pleasant, South Carolina

## Abstract

**Background:**

The BIOFIRE® Blood Culture Identification 2 (BCID2) Panel (bioMérieux) allows for rapid detection of pathogens and antibiotic resistance genes in bloodstream infections (BSIs), including CTX-M, an Extended Spectrum Beta-Lactamase. Atrium Health’s treatment algorithm recommends cefepime (FEP) over ceftriaxone (CRO) for BSI due to non-CTX-M *Escherichia coli*, *Klebsiella pneumoniae* group, *Klebsiella oxytoca*, or *Proteus* spp. in critically ill patients. It is unclear if use of FEP improves outcomes over CRO in this setting.

**Methods:**

This was a multisite, retrospective cohort study of adults with BSI due to *E. coli*, *K. pneumoniae* group, *K. oxytoca*, and *Proteus* spp. without CTX-M detected by BCID2 from April 4, 2022 – February 12, 2023. The primary outcome was a composite of in-hospital mortality and length of stay (LOS) more than 14 days in patients who received FEP vs CRO. Secondary outcomes included in-hospital mortality, hospital LOS, intensive care unit (ICU) LOS, rates of mechanical ventilation, and rates of CRO and FEP resistance.

**Results:**

Three hundred patients were enrolled: 150 in each group. Patients in the FEP group were more severely ill at baseline by ICU status and PITT bacteremia score and were less likely to have a urinary source of BSI (Table 1). The composite primary outcome of in-hospital mortality or extended LOS occurred in 54/150 (36%) in the FEP group vs 24/150 (16%) in the CRO group; (p < 0.001). In-hospital mortality, hospital LOS, rates of mechanical ventilation and CRO resistance were higher in the FEP 5/158 (3%) vs. 2/150 (1%) in the CRO group. ICU LOS, in-hospital mortality and rates of FEP resistance were similar (Figures 1 and 2). Of note, of the 7/308 isolates with CRO resistance there was no in-hospital mortality or extended LOS in the CRO group and 5/7 were Proteus spp. There was no FEP resistance.

**Table 1.**
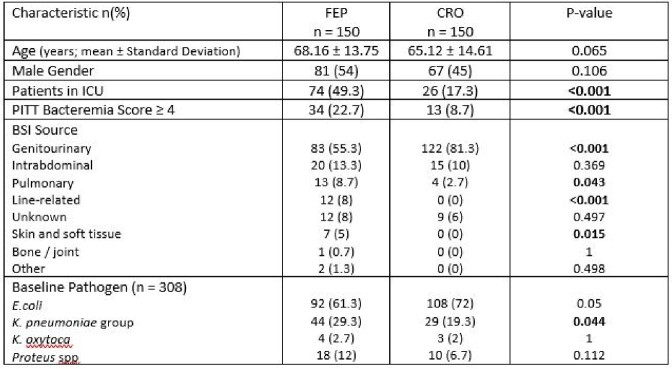
Select Baseline Characteristics

**Figure:**
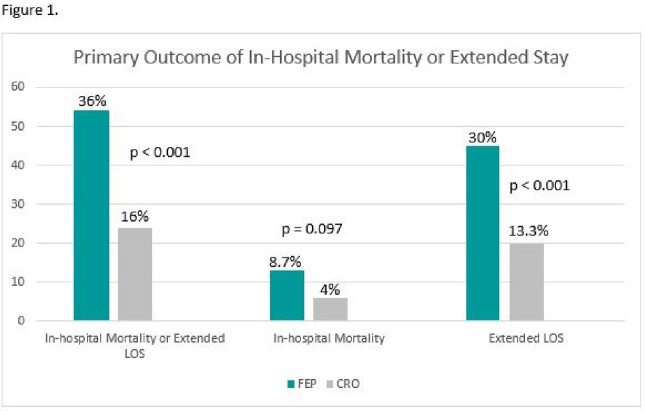

**Figure:**
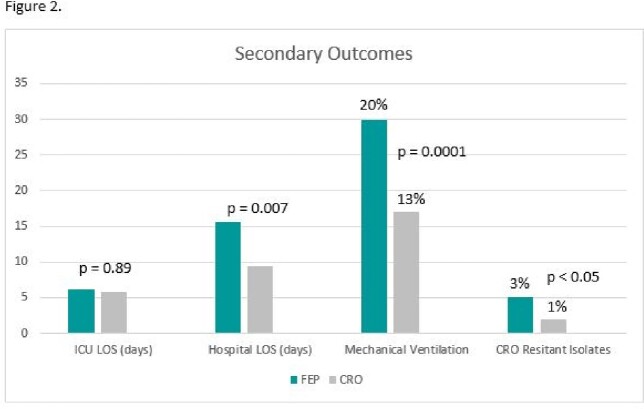

**Conclusion:**

Though CRO resistance was relatively low, it was most common among *Proteus* spp. Treatment with FEP was not associated with improved clinical or microbiological outcomes overall compared to CRO. These results highlight potential overuse of FEP in BSIs due to *E.coli, K. pneumoniae* group, or *K. oxytoca* and may have implications on the selection of empiric therapy.

**Disclosures:**

**All Authors**: No reported disclosures

